# Intraoperative Finding of Neurocysticercosis in a Patient with a Fourth Ventricular Mass

**DOI:** 10.4269/ajtmh.13-0441

**Published:** 2014-07-02

**Authors:** Michael J. Kavanaugh, W. Christopher Fox, Ryan C. Maves

**Affiliations:** Division of Infectious Diseases, Naval Medical Center San Diego, San Diego, California; Department of Neurological Surgery, Neurological Institute, Columbia University College of Physicians and Surgeons, New York, New York

**Figure 1.**
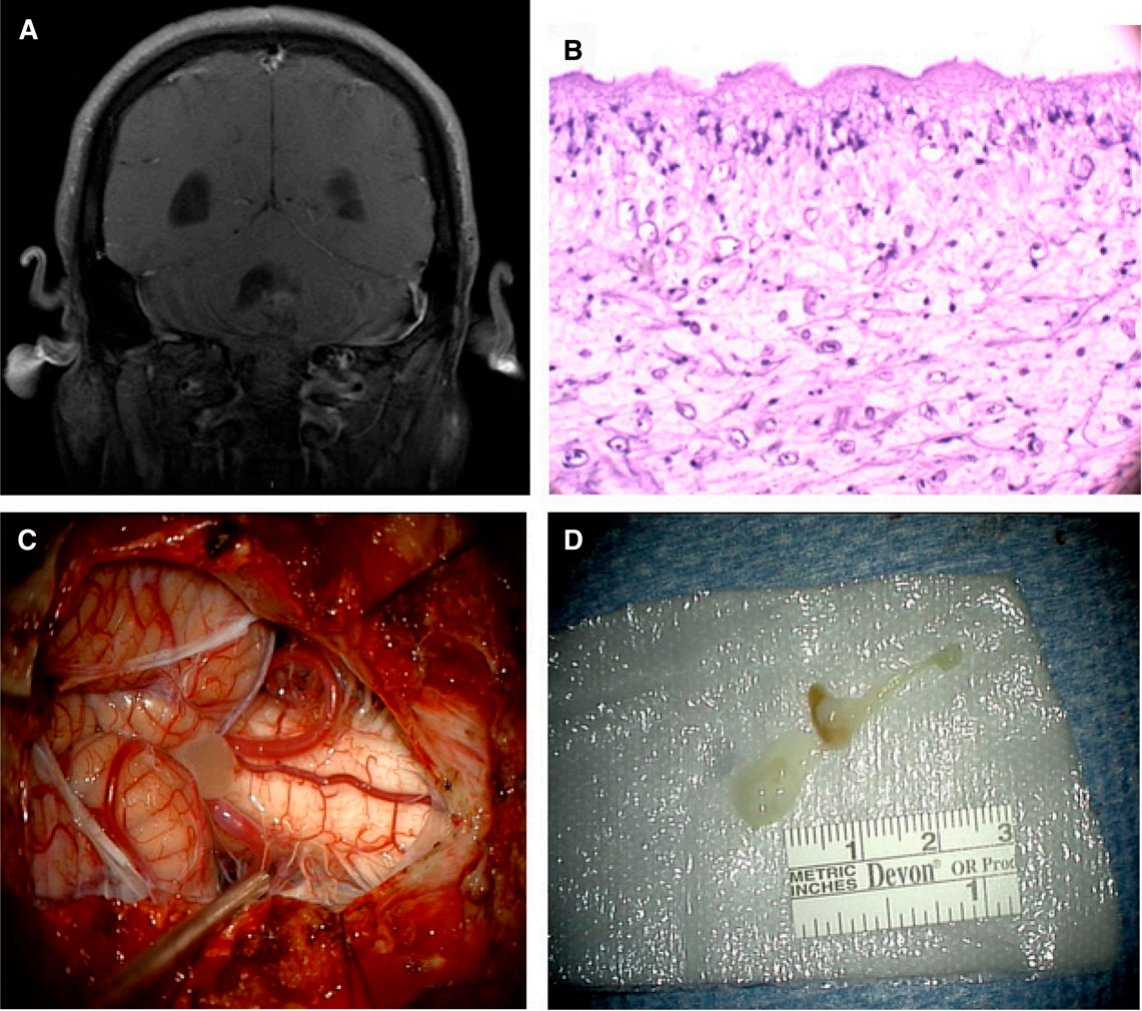
(**A**) Gadolinium-enhanced magnetic resonance imaging (MRI) of the brain showing evidence of a fourth ventricular cystic structure. (**B**) Histopathology of a surgically removed cyst with i) cuticular layer with microvilli, ii) cellular layer, and iii) reticular layer. (**C**) Dissected cyst (neurocysticercosis) protruding from fourth ventricle. (**D**) Cyst post-excision.

**Figure 2.**
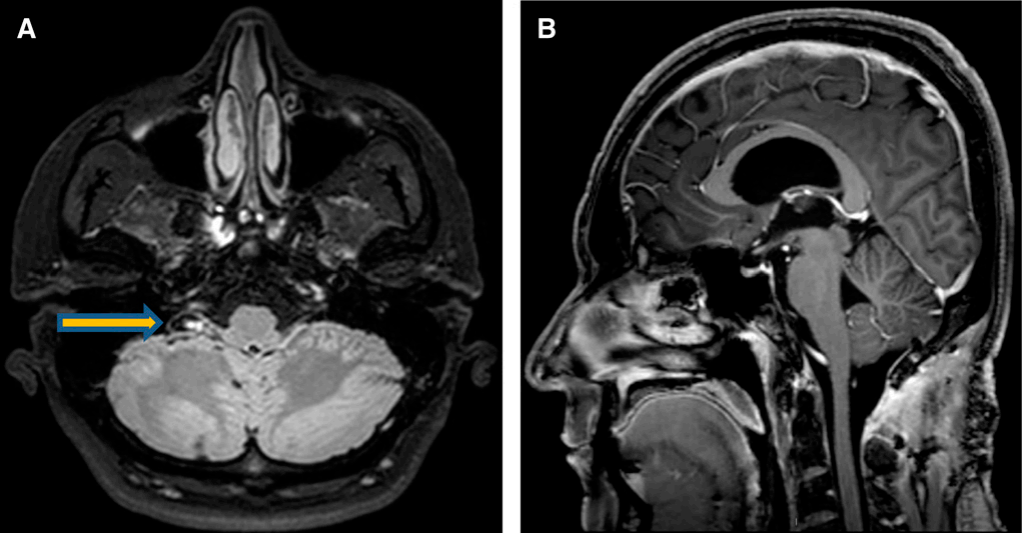
Axial T2. Follow-up imaging 2 months post-surgical. (**A**) 1.2 cm cyst located at the right Foramen of Luschka (lateral recess of the fourth ventricle) consistent with neurocysticercosis cyst with internal scolex. (**B**) Post-surgical image displaying post-surgical changes to the fourth ventricle without evidence of residual cyst.

A 33-year-old male presented for resection of an intracranial mass after 1 year of recurrent headaches and dizziness. Cerebrospinal fluid testing had shown a lymphocytic pleocytosis (163 cells/mm^3^, 59% lymphocytes, 20% neutrophils) with normal protein (42 mg/dL), glucose (54 mg/dL), cultures, and serologic testing. Multiple brain magnetic resonance imaging (MRI) studies showed enlarged ventricles but no masses. Immediately before surgery, a discrete cystic structure was visible on imaging (Figure 1

). A large cyst consistent with neurocysticercosis was identified intraoperatively. No additional lesions were observed, and he had complete resolution of symptoms. He received 7 days of albendazole, dexamethasone, and levetiracetam per guidelines for a solitary lesion.^1^ Follow-up imaging using higher resolution (1 mm versus 6 mm planes) and volumetric sequencing displayed an extra-parenchymal cyst with a visible scolex lateral to the fourth ventricle (Figure 2

). As a result of surgical excision, there was no evidence of a residual fourth ventricle cyst. His MRI spine was negative, and he received two additional months of therapy until radiographic resolution, per guidelines for extra-parenchymal lesions.^1^ This thin walled extra-parynchymal cyst was uncovered with higher quality imaging using both finer planes and an ultrafast MRI technique known as volumetric imaging (e.g., fast imaging employing steady-state acquisition [FIESTA] sequencing). Ideally, higher quality imaging would have revealed these cysts preoperatively. However, his films were suggestive of mass lesion and thus volumetric imaging was not performed until the surgical pathology provided a definitive diagnosis. This case displays the role that high quality volumetric imaging plays in the diagnosis of neurocysticercosis.
